# Adropin exerts neuroprotection in an experimental rat model of Parkinson’s disease 

**DOI:** 10.22038/ijbms.2025.82498.17830

**Published:** 2025

**Authors:** Ayse Ozkan, Hande Parlak, Osman Sinen, Mehmet Bulbul, Mutay Aydin Aslan, Aysel Agar

**Affiliations:** 1 Department of Physiology, Akdeniz University, Faculty of Medicine, Antalya, Turkey; 2 Department of Physiology, Izmir Bakırçay University, Faculty of Medicine, İzmir, Turkey; 3 Department of Physiology, Amasya University, Faculty of Medicine, Amasya, Turkey; 4 Department of Medical Biochemistry, Faculty of Medicine, Antalya, Turkey

**Keywords:** Adropin, Dopamine, GPR19, Parkinson’s disease, VEGFR2

## Abstract

**Objective(s):**

This study was planned to elucidate the mechanism of the protective effect of adropin in an experimental rat model of Parkinson’s Disease (PD).

**Materials and Methods:**

Three-month-old male Wistar rats were randomly divided into four groups: i) Control, ii) Sham, iii) PD, and iv) PD+Adropin. The performance tests were performed seven days after the 6-Hydroxydopamine hydrochloride (6-OHDA) injection into the striatum. The immunoreactivities for tyrosine hydroxylase (TH), G protein-coupled receptor 19 (GPR19), and vascular endothelial growth factor receptor 2 (VEGFR2) were detected by immunohistochemistry (IHC) in the substantia nigra (SN). Dopamine levels were measured by mass spectrometry. Glycogen synthase kinase 3β (GSK3β) and pGSK3β (Ser9) protein levels were evaluated by western blot analysis.

**Results:**

Our study demonstrated that motor performances were significantly improved by adropin treatment. Central adropin injection prevented the loss of nigral dopaminergic neurons and induced VEGFR2 expression but not GPR19 compared to the PD group. The ratio of p-GSK3β/GSK3β did not differ between groups. However, the level of dopamine in SN was increased with adropin injection in the PD+Adropin group.

**Conclusion:**

Our findings reveal that adropin administration has a protective effect on nigral dopaminergic neurons and acts through the VEGFR2 signaling pathway.

## Introduction

Parkinson’s Disease (PD) is the second most prevalent neurodegenerative disorder after Alzheimer’s Disease (AD), affecting over ten million people worldwide and eventually leading to death (1). There is a progressive loss of neuromelanin-containing dopaminergic neurons in the substantia nigra (SN), resulting in signs and symptoms such as resting tremor, rigidity, akinesia, and postural instability (2). While oxidative stress, mitochondrial dysfunction, and inflammation are among the causes of PD, the pathogenic mechanism of dopaminergic neuron loss remains incompletely understood (3). 

Neuroinflammation is increasingly being recognized as a key factor in the progression of PD (4). α-synuclein and Lewy bodies cause an inflammatory microglial response and stimulate reactive astrocyte types. As a result, neurotoxic chemokines and cytokines such as TNF-α and IL-1β are produced, accelerating the degeneration of nigral dopaminergic neurons (5, 6).

Adropin is a 76-amino acid peptide hormone discovered by Kumar and colleagues in 2008 (7). This small peptide, which is encoded by the Enho gene, controls lipid metabolism and energy homeostasis. In the central nervous system, adropin is expressed in neurons, granular, neuroglial, and Purkinje cells (8, 9). It promotes endothelial nitric oxide synthase (eNOS) and vascular endothelial growth factor receptor 2 (VEGFR2) expression in endothelial cells by activating the phosphatidylinositol 3-kinase (PI3K)/protein kinase B (AKT) and extracellular signal-regulated kinase 1/2 (ERK1/2) intracellular signaling pathways (9, 10). Stein et al. published a paper in 2016 in which they identified an adropin transmembrane receptor named G protein-coupled receptor 19 (GPR19), which is similar to the D2 dopamine receptor family (11). GPR19 receptor is strongly expressed in the cerebellum, caudate, putamen, thalamus, hypothalamus, hippocampus, frontal cortex, and olfactory bulb (12, 13). The researchers noted that overexpression of GPR19 receptors increased ERK and AKT phosphorylation in neuronal cells (14). Both GPR19 and VEGFR2 receptors activate the PI3K/Akt signaling pathway, influencing neuronal life (14-16). GSK3β, a multifunctional serine/threonine kinase, plays important roles in multiple pathways involved in cell metabolism, such as proliferation, apoptosis, and glucose metabolism. Therefore, GSK3β is a critical signaling protein that regulates brain development (17). It is connected to the extracellular environment primarily via PI3K/Akt induced by various environmental or cellular insults to neuronal response (18). GSK3β plays an important role in synaptic plasticity at gamma-aminobutyric acid-ergic (GABAergic) and glutamatergic synapses (19).

Adropin plays a protective role in regulating atherogenesis and cardiovascular diseases (20-22). Serum adropin levels are inversely related to serum homocysteine concentrations, risk factors for cardiovascular and coronary atherosclerosis (22). In addition, adropin modulates the Notch1 signaling pathway, which is mediated by neuronal recognition molecule 3 (NB3). Wong and colleagues have found that adropin controls locomotor activity and motor coordination through the NB3/Notch signaling pathway and plays a crucial role in cerebellar development (23). 

This study aimed to test whether adropin administration affects the 6-OHDA-induced PD model of rats and subsequently determine whether it depends on VEGFR2 or GPR19 receptors and activation of intracellular pathways associated with these receptors, which offers a potential therapy for PD.

## Materials and Methods

### Subjects

Male Wistar rats (280–330 g) were housed under standard laboratory conditions (24 ± 2 °C; 12/12 hr light-dark cycle; 50 ± 5 % humidity) obtained from Akdeniz University Laboratories (Akdeniz University Laboratories, Antalya, Turkey). During the experiments, they were given unlimited access to food and water. All surgical and behavioral procedures were carried out in accordance with the Institutional Animal Care and Use Committee guidelines at Akdeniz University Faculty of Medicine, Antalya, Turkey (B.30.2.AKD.0.05.07.00/41). Moreover, all experimental procedures followed the National Institutes of Health Guide for the Care and Use of Laboratory Animals (NIH Publications No. 8023, revised 1978). All efforts were made to minimize animal suffering and reduce the number of animals used.

### Experimental design

Rats were randomly divided into four groups as: 

(i) **Control **(0.9% saline containing ascorbic acid (the solvent of 6-OHDA) was applied via bilateral stereotaxic injection into the striatum), (ii) Sham (received 0.9% saline (the solvent of adropin) into the lateral ventricle via stereotaxic injection), (iii) PD (received 3 µl of 6-OHDA (12 µg/µl) via bilateral stereotaxic injection into the striatum), (iv) PD + Adropin (received 0.9% saline containing adropin (1 nmol) into the lateral ventricle and 3 µl of 6-OHDA (12 µg/µl) via bilateral stereotaxic injection into the striatum). 

**Figure 1:**
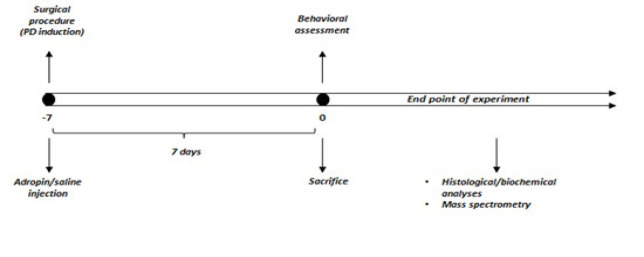
Schematic illustration of the experimental design

On the seventh day following stereotaxic surgery, motor activity tests were performed, animals were sacrificed, and brain samples were collected for biochemical and histological analysis. Figure 1 represents the experimental protocol in detail. 

### Surgery

Rats were anesthetized intraperitoneally (IP) with ketamine (60 mg/kg) and xylazine (12 mg/kg) and immediately placed on a stereotaxic frame. 6-OHDA was injected bilaterally into the striatum at the following coordinates: 0.7 mm anteroposterior (AP), 3.4 mm mediolateral (ML) to the bregma, and 5 mm dorsoventral (DV) from the dura to create a 6-OHDA-induced PD model. The lesion was created by a single injection of 6-OHDA (12 µg/µl at a flow rate of 1 µl/min) dissolved in 0.1% ascorbic acid solution with 0.9% saline (24). 

Adropin was dissolved in 0.9% saline and administered into the lateral ventricle at the following coordinates: AP, -0.8 mm; ML, -1.4 mm; DV, 2.2 mm from the bregma, according to the atlas of Paxinos and Watson (25). The skin was closed with a 4.0 silk suture, and meloxicam (1 mg/kg, IP) was injected daily for two postoperative days, as described previously (26). 

### Behavioral analysis

*Assessment of locomotor performance: To evaluate *locomotor activity, the animals were placed in an open field area and tested for five minutes, as previously described (27, 28). Before placing another animal, the open field was cleaned with 10% ethanol to attenuate odors. The movement was recorded and expressed as an ambulatory activity using a locomotor activity system (MAY 9908-Activity Monitoring System-Commat, Turkey).

*Motor coordination and balance*

On the seventh day following lesion surgery, an accelerated rotarod test was performed with a rotarod apparatus (Ugo Basile) to assess motor coordination and balance (26). Briefly, all rats were subjected to a 3-day training program until they achieved a stable baseline level of performance. During this period, rats were trained to walk against the rotating drum at a constant speed of 12 R.P.M. (revolutions per minute) for a maximum of two minutes. Four training trials were conducted per day, one hour apart. Rats that fell during a training trial were placed back on the rotating drum. Rats were placed on the horizontal rotating drum, and the latency to fall was recorded by measuring the latency for five minutes using an accelerating speed (4 to 40 R.P.M.) (29). 

*Catalepsy test*

Vertical wire netting (56.5 x 23.5 cm; mesh 1 x 1 cm; diameter 2 mm) was used to determine the state of catalepsy. The rats were placed on the wire net with all paws on, and the time it took for at least one paw to be actively displaced from the bar was recorded (30). Each rat was tested five times on the grid, with the average of the five values used to calculate the descent latency time.

### Tissue collection

Rats were anesthetized with urethane (1 g/kg, IP) seven days after lesion surgery and transcardially perfused with heparinized phosphate-buffered saline (PBS; pH: 7.4) for biochemical analysis. The brains were prepared for immunofluorescence labeling with saline, followed by 4% paraformaldehyde (PFA) in PBS. Before each biochemical analysis, protein concentrations were measured at 595 nm using a modified Bradford assay (31).

### Quantitative mass spectrometric measurement of dopamine

Sigma-Aldrich (St. Louis, MO, USA) provided the standards for dopamine (DA). To make standard solutions of DA, 0.01 g of each compound was weighed into a 10 ml glass tube. Then, 1 ml of 98–100% formic acid (Sigma-Aldrich, St. Louis, MO, USA) and 9 ml of LC-grade water were added. As previously described, an optimized multiple reaction monitoring (MRM) method was developed using ultra-fast liquid chromatography (UFLC) coupled with tandem mass spectrometry (MS/MS) (32). A UFLC system (LC-20 AD UFLC XR, Shimadzu Corporation, Japan) was connected to a triple quadrupole mass spectrometer (LCMS-8040) (Shimadzu Corporation, Japan). Chromatographic separations were performed on an HPLC column (Inertsil ODS-4, 3 x 100 mm, 2 µm, GL Sciences Inc. Tokyo, Japan) kept at 25 °C. Gradient elution with a 0.4 ml/min flow rate was used to detect DA. MRM transitions and responses in positive electrospray ionization were automatically optimized for DA (ESI). The responses to DA were optimized to have a linear calibration range of 50 to 1000 ng/ml and a sample analysis time of 4 min. 

### Measurement of adropin levels in the striatum

The level of adropin in striatal tissues was determined using an Enzyme-linked immunosorbent assay (ELISA) kit (MyBioSource, San Diego, California, USA, Cat. No. MBS760446) according to manufacturer’s instructions. A sufficient amount of tissue supernatant was added to the appropriate microtiter wells, and all samples were assessed in duplicate. A spectrophotometric plate reader was used to measure the optical absorbance at 450 nm. A standard curve was created, and adropin concentrations were calculated using the standard curve. Adropin concentration was expressed as pg/mg tissue protein. 

### Immunoreactivity for VEGFR2, GPR19, and TH in SN

Brains from rats were processed 7 days after the vehicle or 6-OHDA injection. Coronal freezing-microtome sections containing SN were immunostained for TH, GRP19, or VEGFR2. The detailed methods were described previously (26, 33). The primary antibodies used included the following: sheep anti-tyrosine hydroxylase (TH), (ab113, Abcam, Cambridge, UK, 1:1000), mouse anti-VEGFR2 (NB200-208, Novus Biologicals, CO, USA, 10µg/ml) and rabbit anti-GPR19 (bs-13522R, Bioss Antibodies, Boston, MA, USA, 1:200). For immunofluorescent staining, the secondary antibodies were: donkey anti-sheep-Alexafluor 568 and donkey anti-rabbit-Alexafluor 568 (ThermoScientific, Waltham, MA, USA, 1:1000). For immunoperoxidase staining, secondary antibody was biotinylated horse anti-mouse IgG (BA-2000, Vector Laboratories, Peterborough, UK) diluted at 1:1000. The detection complex was Vectastain Elite ABC (PK-6100, Vector Laboratories) and the chromogen reagent was 3,3’-Diaminobenzidine (DAB), (SK-4100, Vector Laboratories). Images of neurons expressing TH and GRP19 were acquired using a fluorescent microscope (Olympus BX43, Tokyo, Japan) equipped with appropriate filters, whereas VEGFR2 immunoreactivity was examined and photographed with a light microscope. The rostra-caudal extent of the nigral sections was located using landmarks from the rat brain stereotaxic atlas. Using x10 or x20 objectives, the number of TH-, GPR19-, and VEGFR2-positive cells in the SNpc were counted.

### Western blot analysis

Striatal protein lysates were prepared using a lysis buffer containing protease and phosphatase inhibitors (Cat. 04906845001; Roche Applied Science) as described before [3]. The total protein concentration was quantified using the Bradford protein assay. 50 µg of protein was separated by SDS polyacrylamide gel, and they were transferred to a nitrocellulose membrane. Membrane blots were blocked with non-fat milk in TBS-T (20 mM TrisCl, pH 7.4; 200 mM NaCl) for 60 min at room temperature, followed by incubation of primary antibodies diluted in blocking solution overnight at 4 °C. Primary antibodies against GSK3β (Cat. #12456), p-GSK3β (Cat.#5558) were from Cell Signaling Technology, and β-actin (Cat. ab6276) was from Abcam. The secondary horse peroxidase antibodies used in this study were anti-rabbit (Cat. A0168) and anti-mouse (Cat. A2074) from Abcam. Immunostained bands were detected by an enhanced chemiluminescence kit (Amersham Biosciences, IL, USA), and densitometric analysis was performed on the scan with ImageJ software programs (NIH, USA). β-actin protein was used as an internal control.

### Statistics

The data were analyzed using Graphpad Prism 7 (Graphpad Software, Inc., USA). Behavioral tests and biochemical parameters were analyzed by One-Way Analysis of Variance (ANOVA) followed by Tukey’s post hoc test for normally distributed variables and Kruskal Wallis followed by Mann-Whitney U test for non-normally distributed variables. The immunoreactivities in different groups were assessed using the Kruskal Wallis and post hoc Dunn tests during immunofluorescence labeling. *P*-values<0.05 were considered significant.

## Results

### Behavioral tests

**Figure 2:**
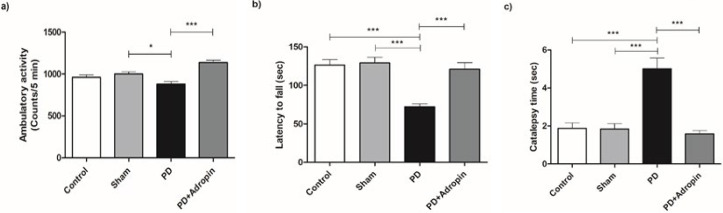
Effect of adropin administration on locomotor activity, motor coordination and catalepsy in male Wistar rats

We used an open-field monitoring system to determine motor performance, and ambulatory activity was recorded for five minutes. The ambulatory activity was significantly decreased in PD group (880.7 ± 29.8 counts/5 min) compared to the Sham group (1001.0 ± 26.6, F = 1.254, R^2 ^= 0.3355, *P*<0.05, Figure 2a). This change was restored by the single administration of adropin in PD model (1137 ± 28.8, F = 1.068, R^2 ^= 0.6800, *P*<0.001, Figure 2a). 

On the seventh day after the 6-OHDA lesion, a rotarod test was performed to assess motor coordination and balance. 6-OHDA lesioned rats exhibited significantly lower latencies (72.20 ± 3.7 sec; *P*<0.001, Figure 2b) than both the control (126.6 ± 6.8; F = 3.348, R^2 ^= 0.7302) and Sham groups (129.2 ± 7.1; F = 3.664, R^2^ = 0.7347). When compared to the PD group, the adropin-administered rats spent significantly more time on the accelerating rotarod (120.9 ± 8.8, F = 5.591, R^2 ^= 0.5886, *P*<0.001, Figure 2b). Similarly, the injection of 6-OHDA significantly enhanced the duration of catalepsy (5.02 ± 0.5 sec) in comparison to the control (1.88 ± 0.2, F = 4.018, R^2^ = 0.5792, *P*<0.001, Figure 2c) and Sham groups (1.84 ± 0.2, F = 3.948, R^2^ = 0.5845,* P*<0.001, Figure 2c). 

### Measurement of dopamine level in substantia nigra

**Figure 3:**
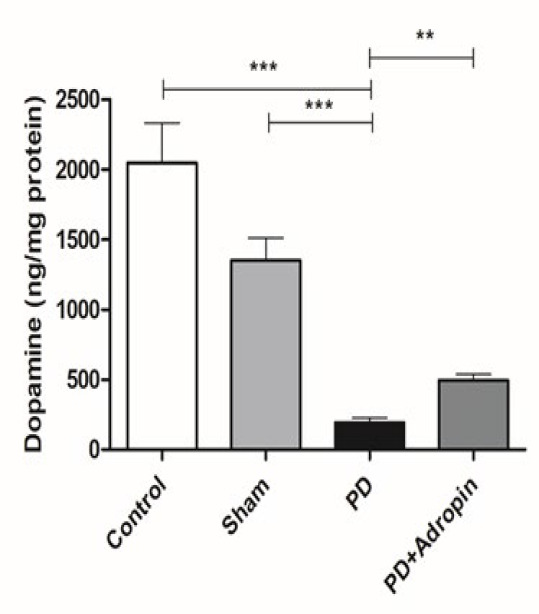
Effect of adropin administration on dopamine levels in male Wistar rats

We used mass spectrometry to measure the dopamine level in the SN after lesion surgery and adropin administration. In comparison to the control (2046 ± 283.6 ng/mg protein, F = 65.67, R^2^ = 0.8083, *P*<0.001) and Sham animals (1350 ± 159.2, F = 20.72, R^2^ = 0.8347, *P*<0.001), 6-OHDA induced a marked reduction in PD groups (191.1 ± 34.9, Figure 3). The 6-OHDA-induced decreases in dopamine levels were prevented by adropin administration (494.1 ± 42.8, F = 1.496, R^2^ = 0.7502, *P*<0.01, Figure 3). 

### Measurement of adropin levels in the striatum

**Figure 4:**
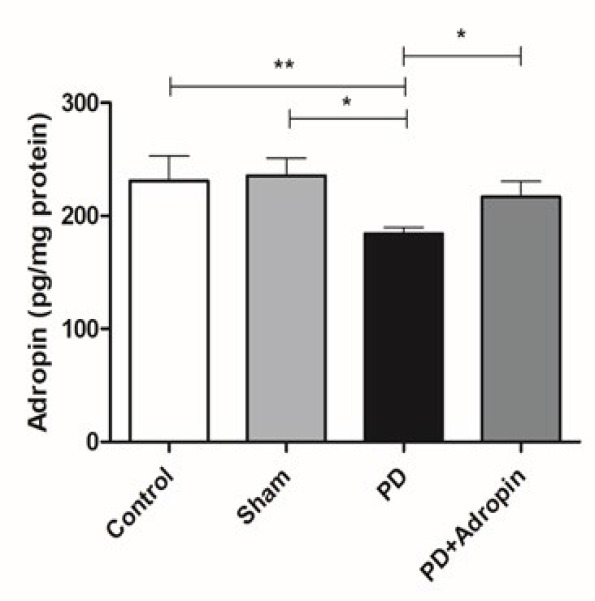
Quantitative ELISA for detection of adropin level in the striatum of male Wistar rats

The adropin levels in the striatum were significantly diminished in PD rats (184.1 ± 5.3 pg/mg protein, Figure 4) compared to control (231.2 ± 21.9, F = 16.56, R^2^ =0.2659, *P*<0.01) and Sham groups (235.6 ± 15.6, F = 8.438, R^2 ^= 0.4455, *P*<0.05). Adropin injection caused a remarkable elevation (216.9 ± 13.8, F = 6.549, R^2^ = 0.2894, *P*<0.05, Figure 4) in the levels of adropin in PD+A group when compared to the 6-OHDA-induced PD group. 

### TH immunoreactivity in nigral substantia nigra

**Figure 5:**
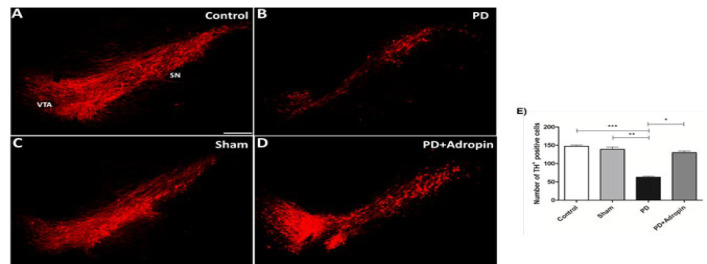
Effect of adropin administration on nigral dopaminergic TH immunoreactivity in 6-OHDA-induced PD model of male Wistar rats (A-D)

TH-positive neurons in the SN were dramatically decreased by 6-OHDA injection in the PD group (62.8 ± 2.7, Figure 5) in comparison to the control (147.2 ± 3.7, F = 1.869, R^2^ = 0.9541, *P*<0.001) and the Sham (139.0 ± 6.1, F = 5.096, R^2^ = 0.8885, *P*<0.001) groups of rats. The administration of adropin significantly increased the number of TH-positive dopaminergic neurons in the nigral section, as shown in Figure 5 (129.9 ± 4.4, F = 2.706, R^2^ = 0.9103). 

### VEGFR2 immunoreactivity in substantia nigra

**Figure 6:**
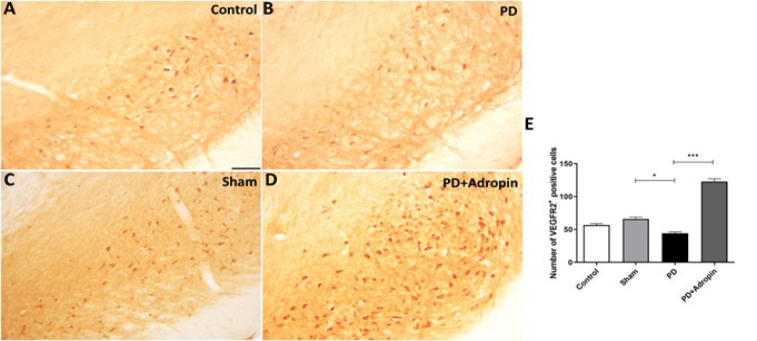
Effect of adropin administration on VEGFR2 immunoreactivity in 6-OHDA-induced PD model in male Wistar rats (A-D)

The VEGFR2-positive neurons in the SN of 6-OHDA-lesioned animals (44.33 ± 2.1, F = 1.718, R^2^ = 0.7905, *P*<0.05, Figure 6) were reduced compared with Sham group (66.0 ± 2.8). We observed that PD rats injected with adropin caused an enhancement in several VEGFR2-positive neurons (122.8 ± 4.4, F = 4.229, R^2^ = 0.9626, *P*<0.001). 

### GPR19 immunoreactivity in substantia nigra

**Figure 7:**
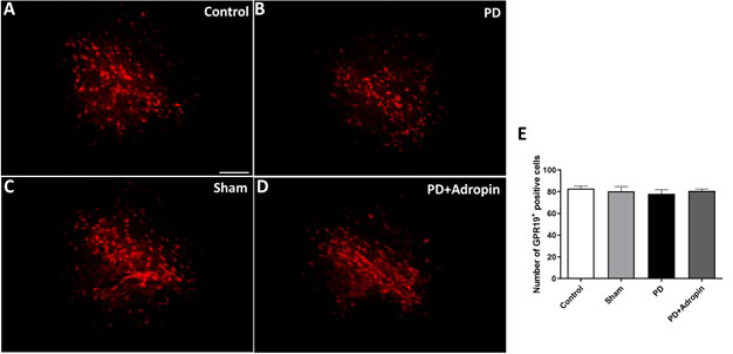
Effect of adropin administration on GPR19 receptor immunoreactivity in 6-OHDA-induced PD model in male Wistar rats (A-D)

No significant difference was observed in the number of GPR19-positive neurons in the PD group (78.0 ± 3.8, *P*>0.05) compared to the control group (82.8 ± 2.3, Figure 7). In addition, the administration of adropin did not affect the numbers of GPR19 positive cells in the SN in comparison to the 6-OHDA-induced PD model (80.71 ± 1.7, *P*>0.05). 

### Expression of GSK3β and p-GSK3β in striatal tissues

**Figure 8:**
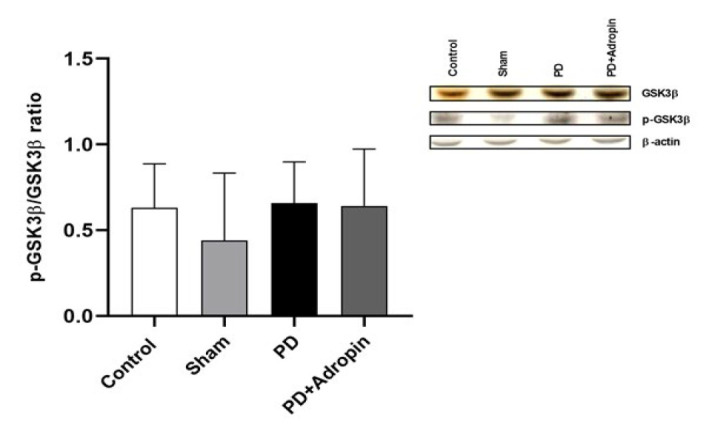
Expression of GSK3β and p-GSK3β protein in the striatum of male Wistar rats as revealed by western blot analysis

To determine whether the administration of adropin could affect the expression of GSK3β and p-GSK3β, we analyzed the expression levels using western blot. As shown in Figure 8, the levels of GSK3β and p-GSK3β did not differ between groups (*P*>0.05).

## Discussion

This study utilized the 6-OHDA-induced experimental rat PD model. By administering adropin to the lateral ventricle, the study revealed a restoration of motor function and protection of dopaminergic neurons in PD.

Various neurotoxins induce PD, including 6-OHDA, maneb, rotenon, paraquat, and MPTP (34). 6-OHDA is the hydroxylated form of the natural neurotransmitter dopamine. In this study, the 6-OHDA rat model was used to lesion the nigrostriatal dopaminergic system as a model of PD. 6-OHDA is not able to cross the blood-brain barrier and should, therefore, be administered directly to the brains of experimental animals via stereotaxic surgery (35). 6-OHDA is preferentially injected stereotaxically into the striatum, the SN, or medial forebrain bundle (MFB) in PD experimental rodent models to disrupt nigral dopaminergic neurons. After injection, 6-OHDA is transferred into dopaminergic neurons by the dopamine transporters (DAT). It then accumulates in dopaminergic neurons, exerting its harmful effects via complex interacting mechanisms involving oxidative stress and mitochondrial defects caused by inhibiting respiratory chain enzyme complexes I and IV (36, 37). Striatal injection of 6-OHDA results in more than 90% depletion of dopamine (36). In our rodent model, 6-OHDA was also injected into the striatum.

Kumar et al. discovered in 2008 that adropin is encoded by the ENHO gene located on chromosome 9p13.3 in mice. They also clarified that adropin has 76 amino acids and was initially described as a secreted peptide (10). Intracerebroventricular injection of adropin (1 nmol) inhibits water drinking with the G-protein-coupled receptor, GPR19 (11). In the present study, the same dose of adropin was also administered to rats intracerebroventricularly. 

Locomotor activity and motor coordination are known to be impaired in 6-OHDA-induced experimental PD models (27). The test was performed on the seventh day after 6-OHDA administration to the striatum of rats bilaterally to determine locomotor activity. Our findings indicated that ambulatory activity was impaired in the PD group. However, administration of adropin improved locomotor performance in the PD model of rats. The duration of time the animals stood on the accelerating rotating rod was measured by the rotarod test, which is used to determine motor coordination and balance (24). The current finding revealed that the duration time on the rotating bar was significantly reduced in the PD group, whereas adropin restored motor coordination in a 6-OHDA-induced PD model. A previous study reported that adropin regulates locomotor activity and motor coordination during nervous system development (23). Besides, it has also been found that in adropin knockout mice, motor coordination is impaired with the weakening of the synaptic connections in the cerebellum (23). Our previous study found enhancement in locomotor activity following the adropin administration (38). The results of the analyses indicated, in line with past research, that the adropin administration enhanced the ambulatory activity and motor performance in a 6-OHDA-induced PD model.

Catalepsy is a well-known symptom of PD, and it is determined by using the classic bar and wire test (39). The time it took to displace one of the paws (descent latency) actively was recorded in the wire test and used to evaluate the degree of catalepsy in the present study. In a study by Rodriguez et al., 6-OHDA-induced catalepsy was observed one day after the lesion and lasted three months (40). Meanwhile, catalepsy is linked to dopaminergic neuron degeneration and decreased dopamine levels in the striatum and SN (40). The current study found that adropin administration reduced catalepsy time, increasing dopamine levels and TH+ cell numbers in SN. Also, following adropin treatment, we observed an increase in striatal adropin levels in the PD group.

Adropin is a key mediator in cell survival and proliferation, and it binds to various receptors, including VEGFR2, GPR19, and NB3 (9, 23, 41, 42). VEGF has a neuroprotective effect on dopaminergic neurons in both *in vitro *and *in vivo* PD models. (43). Besides, Herran and colleagues reported that *in vivo *VEGF administration enhanced neuroregeneration in 6-OHDA-lesioned rats in the SN (44). In a previous study, treatment with VEGF-B has been shown to increase the number of TH-positive cell bodies in the SN (45). In addition, when VEGFR2 is activated, the AKT and ERK pathways are also activated in the cell (46). In this study, central adropin administration elevated adropin levels in the striatum, and the expression of VEGFR2 was dramatically increased in the adropin-treated group. Adropin may have protected dopaminergic neurons in the SN by binding to the VEGFR2 receptor. Hence, the improvement in dopamine levels and motor performance observed in the present study might be attributed to the preservation of dopaminergic neurons in the SN after adropin administration in rats. 

GSK3β is tightly related to the loss of dopaminergic neurons in the PD model of animals, and its inhibition can protect dopaminergic neurons from toxicity (47). It can be inactivated by Akt and several protein kinases such as PKA, PKB, and p90RSK by phosphorylation of N terminal single serine residue (Ser9) (48-51). It is known that GSK3β has a crucial role in some neurodegenerative and neuropsychiatric diseases (52-54). However, there are many conflicting results regarding whether SN or striatal GSK3β levels are increased, decreased, or unchanged in experimental PD (55-58). Our previous study argued that the protective effect of exercise by aging with the increase of adropin is not revealed by the GSK3β signaling pathway (59). However, in another previous study, researchers reported that adropin treatment enhanced the GSK3β phosphorylation in the liver (60). Thus, our analysis clearly shows that 6-OHDA administration did not affect the phosphorylation of GSK3β in the SN regions of the PD group of animals compared to the control. The results of our study show that adropin is a potential neuroprotectant on dopaminergic neurons in PD but obviously does not exert this protective effect through the GSK3β. Therefore, the pathway through which it exerts this favorable effect should be addressed in further investigations. 

Parkinson's disease (PD) was induced via surgical intervention on day -7, followed by the administration of adropin or saline. Behavioral assessments were conducted on day 0, after which the animals were euthanized. Subsequently, histological and biochemical analyses, as well as mass spectrometry, were performed

a) Ambulatory activity, b) Latency to fall, c) Catalepsy time. PD: Parkinson’s Disease. For each group, n=10 and data are expressed as mean ± SEM. All significant differences are shown as *P<0.05, ***P<0.001.

For each group, n=6, and data are presented as mean ± SEM. Significant differences were shown as **P<0.01, ***P<0.001. PD: Parkinson’s disease

For each group, n=7, and data are expressed as mean ± SEM. Significant differences were shown as *P<0.05, **P<0.01. PD: Parkinson’s disease

The scale bar represents 100 µm. The quantitative analysis of TH-positive neurons in SN. *P<0.05, **P<0.01, ***P<0.001. n=3 rats per group. TH: Tyrosine Hydroxylase, PD: Parkinson’s Disease, 6-OHDA: 6-hydroxydopamine

The scale bar represents 100 µm. The quantitative analysis of VEGFR2-positive neurons in SN. *P<0.05, ***P<0.001. n=3 rats per group.

VEGFR2: Vascular Endothelial Growth Factor Receptor 2, 6-OHDA: 6-hydroxydopamine, PD: Parkinson’s Disease

The scale bar represents 100 µm. The quantitative analysis of GPR19-positive neurons in SN. n=3 rats per group. GPR19: G-protein-coupled receptor 19, 6-OHDA: 6-hydroxydopamine, PD: Parkinson’s Disease

Data are expressed as mean ± SEM (n=3). GSK3β: Glycogen synthase kinase-3 beta

## Conclusion

The current study found that adropin improves motor coordination and locomotor activity in an experimental 6-OHDA-induced model of PD. Central adropin administration increased dopamine levels and VEGFR2 expression in the SN and prevented nigral dopaminergic neuron death in the PD model of rats. This study also demonstrated that the GPR19 receptor is located in the SN for the first time. 

Overall, this study provides new information that adropin may have a potentially protective effect in PD and be considered a valid therapeutic agent. Indeed, future studies are required to characterize the protective mechanisms afforded by adropin.

## Declaration

We have not used any AI tools or technologies to prepare this manuscript.
